# Supplementary material for: Bodily expressed emotion understanding through integrating Laban movement analysis
Source: Patterns (N Y). 2023 Aug 22;4(10):100816. doi: 10.1016/j.patter.2023.100816 (PMC10591137; doi:10.1016/j.patter.2023.100816)
Supplement: Table S1. AP values for all emotion categories on the BoLD validation set [file mmc1.pdf]

Patterns, Volume *4*

Supplemental information

Bodily expressed emotion understanding through integrating Laban movement analysis

Chenyan Wu, Dolzodmaa Davaasuren, Tal Shafir, Rachelle Tsachor, and James Z. Wang

Table S1
AP values for all emotion categories on the BoLD validation set
To provide a comprehensive view of how LMA improves BEEU across all emotion categories, we present a category-specific performance analysis of MANet in this table. Consistent with Tables 8 and 9 in the main body of the text, "Baseline-1" represents the original V-Swin model without the dual-branch and fusion elements, essentially operating without the assistance of LMA labels. "Baseline-2" refers to the MANet model excluding the Bridge loss. Our final model is denoted as "Ours". For each emotion category, the most effective and second most effective approaches are highlighted with color indicators. The table illustrates that, out of 26 categories, our model outperforms in 16. Even in the remaining categories, our model's performance is not substantially inferior to either Baseline-1 or Baseline-2. Consequently, it can be inferred that integrating LMA labels from the BoME dataset and utilizing our proposed network model not only amplifies the accuracy of predicting happiness and sadness, as stated in the main text, but also significantly improves the prediction of various other emotions with negligible adverse effects on the remaining categories.

Emotion Category	Baseline-1	Baseline-2	Ours
Peace	27.59	29.73	30.49
Affection	30.69	30.11	33.16
Esteem	17.43	16.08	15.22
Anticipation	29.51	29.59	28.86
Engagement	44.67	46.57	44.35
Confidence	41.22	43.68	44.04
Happiness	44.97	44.41	45.76
Pleasure	28.12	31.56	30.11
Excitement	30.36	28.62	30.77
Surprise	11.53	13.24	14.89
Sympathy	11.49	13.36	16.42
Confusion	23.44	22.93	22.49
Disconnection	10.11	10.22	10.63
Fatigue	16.89	14.61	17.21
Embarrassment	3.22	3.55	3.80
Yearning	7.70	3.49	3.82
Disapproval	14.41	17.33	17.92
Aversion	10.00	10.73	9.54
Annoyance	18.81	20.48	19.44
Anger	14.90	15.15	14.00
Sensitivity	10.75	11.40	14.08
Sadness	17.91	17.60	26.66
Disquietment	21.94	22.53	21.06
Fear	17.09	17.46	18.49
Pain	4.44	4.61	5.97
Suffering	10.00	12.08	13.26
Mean	19.97	20.43	21.25
