# Supplementary material for: Bodily expressed emotion understanding through integrating Laban movement analysis
Source: Patterns (N Y). 2023 Aug 22;4(10):100816. doi: 10.1016/j.patter.2023.100816 (PMC10591137; doi:10.1016/j.patter.2023.100816)
Supplement: Document S2. Article plus supplemental information [file mmc2.pdf]

Patterns

Article

Bodily expressed emotion understanding through integrating Laban movement analysis

Graphical abstract

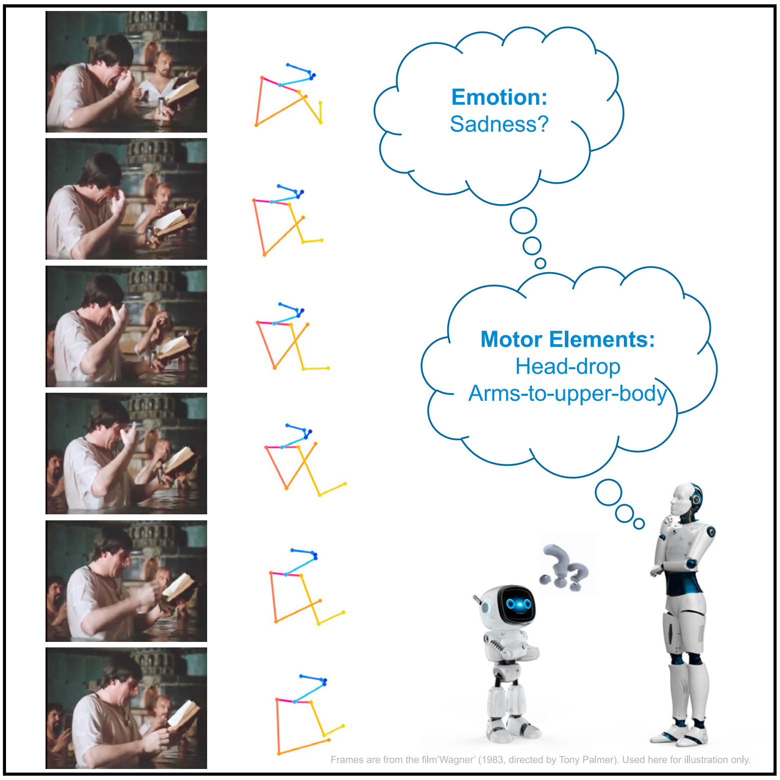

Highlights

- BoME dataset characterized by Laban movement analysis
- Estimating Laban movement analysis motor elements with deep neural networks
- Jointly training dual-branch, dual-task movement analysis network
- State-of-the-art performance on both motor element and emotion recognition tasks

Authors

Chenyan Wu, Dolzodmaa Davaasuren, Tal Shafir, Rachelle Tsachor, James Z. Wang

Correspondence

jwang@ist.psu.edu

In brief

This study uses human motor elements to better understand emotions expressed through body movements, a key aspect of human communication. The authors built a comprehensive BoME dataset and developed a dual-source model for automated emotion recognition. This approach significantly enhances machine perception of emotions, promising advancements in human-machine interaction, robotics, and mental health diagnostics. The integration of insights from computing, psychology, and performing arts could revolutionize our understanding of non-verbal communication in various professional and social contexts.

Wu et al., 2023, Patterns *4*, 100816
October 13, 2023 © 2023 The Authors.
https://doi.org/10.1016/j.patter.2023.100816

CellPress

Article

Bodily expressed emotion understanding through integrating Laban movement analysis

Chenyan Wu,[1] Dolzodmaa Davaasuren,[1] Tal Shafir,[2] Rachelle Tsachor,[3] and James Z. Wang[1,4,5,*]
[1]Data Science and Artificial Intelligence Area, College of Information Sciences and Technology, The Pennsylvania State University, University Park, PA 16802, USA
[2]The Emili Sagol Creative Arts Therapies Research Center, University of Haifa, Haifa 3498838, Israel
[3]School of Theatre and Music, University of Illinois, Chicago, IL 60607, USA
[4]Human-Computer Interaction Area, College of Information Sciences and Technology, The Pennsylvania State University, University Park, PA 16802, USA
[5]Lead contact
*Correspondence: jwang@ist.psu.edu
https://doi.org/10.1016/j.patter.2023.100816

THE BIGGER PICTURE Body movements carry important information about a person's emotions or mental state and are essential in everyday communication. Enhancing machines' ability to understand emotions expressed through body language can improve communication between assistive robots and children or elderly users, provide psychiatric professionals with quantitative diagnostic and prognostic assistance, and bolster safety by preventing mishaps in human-machine interactions. This study develops a high-quality human motor element dataset based on the Laban movement analysis movement coding system and utilizes that to jointly learn about motor elements and emotions. Our long-term ambition is to integrate knowledge from computing, psychology, and performing arts to enable automated understanding and analysis of emotion and mental state through body language. This work serves as a launchpad for further research into recognizing emotions through the analysis of human movement.

1 2 3 4 5 **Proof-of-Concept:** Data science output has been formulated, implemented, and tested for one domain/problem

SUMMARY

Bodily expressed emotion understanding (BEEU) aims to automatically recognize human emotional expressions from body movements. Psychological research has demonstrated that people often move using specific motor elements to convey emotions. This work takes three steps to integrate human motor elements to study BEEU. First, we introduce BoME (body motor elements), a highly precise dataset for human motor elements. Second, we apply baseline models to estimate these elements on BoME, showing that deep learning methods are capable of learning effective representations of human movement. Finally, we propose a dual-source solution to enhance the BEEU model with the BoME dataset, which trains with both motor element and emotion labels and simultaneously produces predictions for both. Through experiments on the BoLD in-the-wild emotion understanding benchmark, we showcase the significant benefit of our approach. These results may inspire further research utilizing human motor elements for emotion understanding and mental health analysis.

INTRODUCTION

Recognizing human emotional expressions from images or videos is a fundamental area of research in affective computing and computer vision, with numerous applications in robotics and human-computer interaction.[1–5] With the development of the body language dataset (BoLD), a large-scale, in-the-wild dataset for bodily expressed emotion, and the corresponding benchmark deep neural network models,[6] research on emotion recognition has increasingly focused on bodily expressed emotion understanding (BEEU).[7–9]

In contrast to the extensively studied facial expression recognition,[10–14] BEEU aims to automatically recognize emotion expression from body movements. Emotion recognition through body movements presents several advantages over reliance on facial inputs. First, in crowded scenes, a person's facial area may be obscured or lack sufficient resolution, but body movements and

 © 2023 The Authors.
This is an open access article under the CC BY license (http://creativecommons.org/licenses/by/4.0/).

postures can still be reliably detected. Second, research has shown that the body may be more diagnostic than the face for emotion recognition.[15] Third, facial areas may be inaccessible in some applications due to privacy and confidentiality concerns. Fourth, it may be difficult to fake subtle emotions through body movements, whereas facial expressions can often be manipulated. Finally, using body movements as an additional modality can lead to more accurate recognition compared with relying solely on facial images or videos.

Facial expression recognition studies often rely on the facial action coding system (FACS) as an intermediate representation.[3,10,13] This approach involves detecting action units (AUs), which are defined as the movements of specific facial muscles in FACS, and subsequently using these detections to recognize emotions. This method is based on the fact that certain muscles (AUs) contract to produce specific facial expressions, such as the corrugator muscle contracting to frown and express anger.

Similarly, people use particular body muscles and skeletal parts to communicate their emotions. For instance, individuals may touch their heads with their hands when feeling sad, as illustrated in the first example of Figure 1. By describing specific movements common to humans and the motor elements that make up these movements, we can establish the relationship between these motor elements and bodily expressed emotion, mirroring the role of FACS in facial expression recognition.

Compared with facial muscle movements, motor elements are often more readily detectable in a video of a person. In addition, these motor elements are generally more clearly defined, rendering them simpler for AI to recognize. Consequently, motor elements can function as a suitable intermediate representation for BEEU, bridging the gap between low-level movement features (e.g., the velocity of human joints) and emotion category labels.

Although some previous studies on BEEU have incorporated motor elements, there remains a gap in the utilization of deep learning-based methods to construct the comprehensive representation of human motion. One pioneering work by Camurri et al.[16] focused on dance movements and utilized handcrafted features such as quantity of motion and contraction index to represent motion descriptors and expressive cues. The extracted features were then inputted into various classifiers, including multiple regression and support vector machines (SVMs). Subsequent studies followed a similar pipeline, with Luo et al.[6] employing only low-level movement features (e.g., velocity and acceleration of human joints) and subsequently using random forest for emotion classification. Supported by the European Union H2020 Dance Project,[17] Niewiadomski et al.[18] designed handcrafted features to represent the lightness and fragility of human movement, while Piana et al.[19] constructed multiple motion features from the 3D coordinates of human joints and employed linear SVMs for classification. However, these methods did not utilize deep neural networks to extract profound learning representations, and handcrafted features often rely on 3D motion data (i.e., 3D coordinates of human joints) as input, which can only be obtained within a lab-controlled environment, thereby limiting their potential applications. A recent study[20] supported by the European Union H2020 EnTimeMent Project[21] employed a neural network for emotion recognition. However, the method still requires the use of a motion capture system to collect 3D motion data in a lab environment as input. Some other

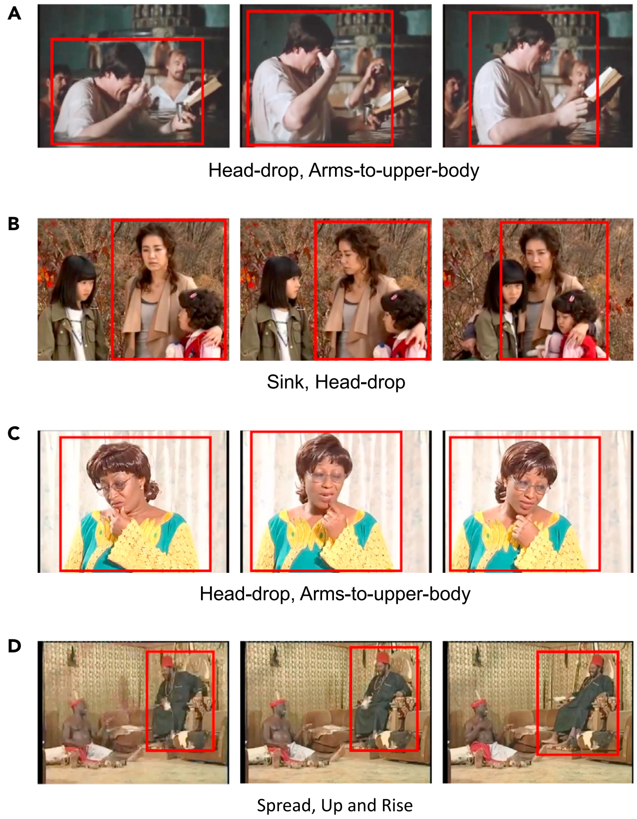

Figure 1. Example video clips from the BoME dataset
Three sample frames are shown for each clip. Instances of interest are bounded by red boxes. The LMA motor elements annotated based on the movement of the person in the red box are shown. Subfigures (A) to (D) incorporate frames from the films "Wagner" (1983, directed by Tony Palmer), "Heaven's Garden" (2011, directed by Jong-han Lee), "REVIVAL - Nigerian Nollywood Movie" (2000), and "The Priest Must Die 2 - Nigerian Nollywood Movie" (2009), respectively.

deep learning-based approaches on BEEU[7–9] utilized techniques developed for video or action recognition, directly feeding human movement videos into a video recognition network and predicting emotion categories without considering the understanding of motor elements.

In this work, we introduce a novel paradigm for BEEU that incorporates motor element analysis. Our approach leverages deep neural networks to recognize motor elements, which are subsequently used as intermediate features for emotion recognition.

A primary challenge in implementing this approach is the limited availability of extensive public image or video datasets suitable for deep learning-based motor element analysis.[3,5,22] To tackle this issue, we created the BoME (body motor elements) dataset, comprising 1,600 high-quality video clips of human movements. We consider different human movements within a single video as distinct clips. Each of these clips is annotated with precise, expert-provided movement labels. We used the AVA video dataset[23] as the video source and applied the Laban movement analysis (LMA) system to describe the motor elements. LMA, which originated within the dance community in the early 20th century, has evolved into an internationally recognized framework for

describing and comprehending human bodily motions. It characterizes human movements into five categories: body, effort, space, shape, and phrasing, and includes over 100 detailed motor elements. To balance the tradeoff between the number of LMA elements and the cost of annotation, we selectively included 11 emotion-related LMA elements. This decision was guided by preliminary psychological research exploring the relationship between emotions and motor elements as described by LMA.[24–27] We designed a systematic procedure for dataset collection and invited a certified movement analyst (CMA), an expert in LMA, to annotate the presence of LMA elements in the human movement clips. Figure 1 shows some examples of the BoME dataset.

Using the established BoME dataset, we examined whether deep neural networks can learn an effective representation of human movement. We deployed several state-of-the-art video recognition networks on BoME to estimate the LMA elements and investigated the impact of factors such as video sampling rate and pretraining datasets on network performance. The results showed that these methods, particularly the Video Swin Transformer (V-Swin),[28] performed well on the BoME dataset, indicating that deep neural networks could learn an appropriate movement representation from BoME.

Finally, we conducted experiments to enhance BEEU by using the BoME dataset as an additional source of supervision. We designed a dual-branch, dual-task network called movement analysis network (MANet), whose branches produce predictions for bodily expressed emotion and LMA labels, respectively. To effectively utilize the movement representation in emotion recognition, we integrated the LMA branch features into the emotion branch. We also introduced a new bridge loss that enables LMA prediction to supervise emotion prediction. Employing a weak supervision strategy, we trained MANet on both the BEEU benchmark BoLD and the BoME datasets. The BEEU results on the BoLD validation and test sets revealed that our approach significantly outperformed all single-task baselines (i.e., approaches that only consider BEEU).

RESULTS

Statistical analysis confirms the effectiveness of the LMA motor elements

To improve the ability of deep neural networks to learn human movement representation and subsequently enhance emotion recognition, we created a high-precision motor element dataset named BoME. This dataset consists of 1,600 human video clips, each expertly annotated with LMA labels. To achieve a balance between precision and utility, we annotated each clip with 11 LMA elements. Research has indicated that these elements are associated with sadness and happiness and are relevant for emotion elicitation and emotional expression, making them valuable for understanding bodily expression.[24–26] In addition, annotating 11 elements is not an overly laborious task for LMA experts, ensuring the quality of the dataset. Table 1 lists the 11 elements and their associated emotions, LMA categories, and descriptions. The experimental procedures provides a comprehensive explanation of our choice of LMA elements and outlines the methodology employed for dataset collection.

For each LMA element, we assigned a five-level label based on the element's duration and intensity in the clip, with level 0 indicating no presence and level 4 signifying maximum presence. The distribution of the five levels for each LMA element is shown in Figure 2. The head-drop element is relatively commonly present in videos, while jump cases are rare. The five-level label can also be interpreted as a binary label, with level 0 representing a negative label and non-zero levels denoting a positive label. On average, each clip in the BoME dataset includes 3.2 positive LMA elements, with a minimum of 1 and a maximum of 10 positive elements per clip.

To confirm the association of the LMA element labels in BoME with specific emotions, the annotator assigned an emotion label to each clip, which was limited to three categories: sadness, happiness, and other emotions. Although emotion recognition studies often require a larger number of emotional categories, we restricted ourselves to these three categories to validate the effectiveness of the LMA element labels.

For each human clip in BoME, we assigned a binary value (0 or 1) to the sadness and happiness emotion categories based on the emotion label, as well as a five-level label (ranging from 0 to 4) for each LMA element. Using these values, we calculated the correlation between LMA elements and the two emotion categories—sadness and happiness—encompassing all the samples marked with these two emotions within the dataset. The correlations are visually depicted in Figure 3. We discovered that sink and head-drop were strongly positively correlated with sadness, while light weight, up, and rise exhibited significant positive correlations with happiness. These results align with previous psychological studies.[24–26] Conversely, jump and rotation did not demonstrate a substantial correlation with either sadness or happiness, which could be attributed to the relatively small sample size for these elements. In addition, based on the emotion labels, we found that instances of sadness accounted for 59.9% of the BoME dataset, while cases of happiness accounted for only 22.2%. This imbalance may stem from the fact that sadness is more easily recognizable to human annotators compared with other emotion categories.

Deep neural networks are capable of estimating LMA motor elements

To examine the potential of deep learning methods to learn an effective representation of human movement, we applied deep neural networks to estimate LMA elements on the BoME dataset. We randomly divided the dataset into a training set consisting of 1,448 samples and a test set containing 152 samples. The original LMA element labels had five levels, but levels 3 and 4 had limited sample sizes, as demonstrated in Figure 2. This posed a challenge for models to accurately estimate the LMA elements. Furthermore, deep neural networks may struggle to precisely determine the duration of each element in a clip, unlike LMA experts. To simplify the task, we treated the LMA element estimation problem as a multi-label binary classification task, with level 0 being designated as a negative label and all non-zero levels as positive labels.

We evaluated the classification performance using two metrics: average precision (AP), or the area under the precision-recall curve, and the area under the receiver operating characteristic curve (AUC-ROC). We reported the mean average precision (mAP) and mean AUC-ROC (mRA) across all categories of LMA elements. Notably, we only used ten elements and excluded the jump element due to the dataset containing an insufficient number of samples (only 11) for jump.

Table 1. The 11 LMA elements coded in the BoME dataset

	LMA element	LMA category	Description
Sadness	passive weight	effort	lack of active attitude toward weight, resulting in sagging, heaviness, limpness, or dropping
	arms-to-upper-body	body	hands or arms touching any part of the upper body (head, neck, shoulders, or chest)
	sink	shape	shortening of the torso and head and letting the center of gravity drop downward, so the torso is convex on the front
	head-drop	body	releasing the weight of the head forward and downward, using the quality of passive weight; dropping the head down
Happiness	jump	body	any type of jumping
	rhythmicity	phrasing	rhythmic repetition of any aspect of the movement, like bouncing, rocking, bobbing, twisting, from side to side, etc.
	spread	shape	when the mover opens his body to become wider
	free flow	effort	lessening movement control, moving like you "go with the flow"
	light weight	effort	moving with a sense of lightness and buoyancy of the body or its parts; gentle or delicate movement with very little pressure and a sense of letting go upward
	up and rise[a]	space/shape	up means going in the upward direction in the allocentric space. Rise means raising the chest up by lengthening the torso
	rotation	space	rotating a body part, or turning the entire body around itself in space, like in a Sufi dance

Psychological studies indicate that the first four elements are associated with sadness and the rest with happiness.
[a]Up and rise are two elements within the space and shape categories, respectively. We follow Shafir et al.[24] and Melzer et al.[25] to merge them into one element because these movements are affined, often occurring together.

As a natural initial attempt, we employed a range of deep learning-based video recognition algorithms to estimate LMA elements from video clips. These algorithms can be categorized based on their input modality as either RGB based or skeleton based. We illustrate the RGB-based and skeleton-based pipeline in Figure 4. We selected four representative video recognition approaches from recent years to benchmark the BoME dataset: temporal segment network (TSN),[29] SlowFast,[30] V-Swin,[28] and PoseC3D.[31] The first three methods are RGB based, while PoseC3D is skeleton based.

The performances of these four algorithms are compared in Table 2. The following outlines the input processing and neural network for each approach.

- TSN[29] partitions a single input clip into multiple sub-clips, selecting one random frame from each. Table 2 employs three sampling rates—the input clip is segmented into 8, 16, or 24 sub-clips, resulting in 8, 16, or 24 frames. For each frame, we cropped the human region from the entire RGB image and resized the area to 224×224 pixels. Region detection was facilitated using OpenPose.[32] The processed frames were subsequently fed into a 2D convolutional neural network. We have chosen the widely used ResNet-50[33] as our 2D convolutional network. Note that while the original TSN incorporates both optical-flow images and RGB images as input, we exclusively used the RGB input without optical-flow to ensure a fair comparison with other RGB-based methods.
- SlowFast[30] samples 32, 48, or 64 frames from the entire input clip, maintaining a temporal stride of 2 between consecutive samples. Consistent with the TSN approach, we cropped the human region from the entire RGB image and resized the area to 224×224 for each frame. The extracted and cropped RGB images were then input into a 3D convolutional network. We adopted a variant of the 3D convolutional ResNet-101[33] as the network, in accordance with the original paper.
- V-Swin[28] employs the same input processing procedure as SlowFast. However, V-Swin utilizes a 3D Transformer network, adapted from the 2D Swin Transformer.[34] We followed the base-Swin setting as outlined in the original paper.
- PoseC3D[31] uniformly samples 48, 72, or 96 frames from the entire input clip. Subsequently, 2D human pose inputs are detected by OpenPose.[32] Finally, a 3D convolutional network processes the human poses and generates predictions. We adopted the network structure provided by MMaction2.[35]

CellPress
OPEN ACCESS

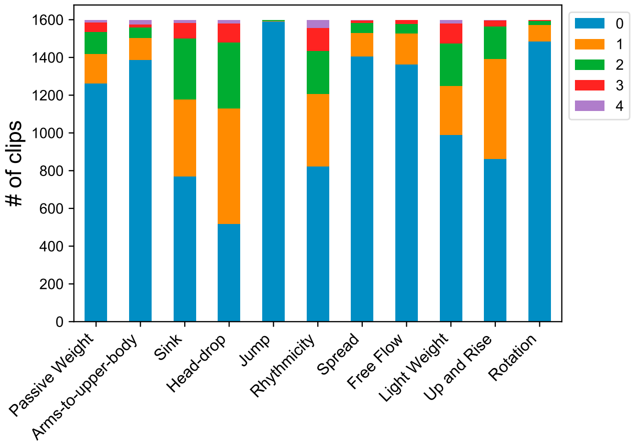

Figure 2. Distribution of the five-level labels for each LMA element

A total of 1,600 video clips have been labeled for 11 LMA motor elements that are considered closely associated with happiness and sadness emotions. Each element is annotated on a scale of zero to four, where zero denotes the absence of the element (represented in blue), and four signifies its maximum presence (represented in purple).

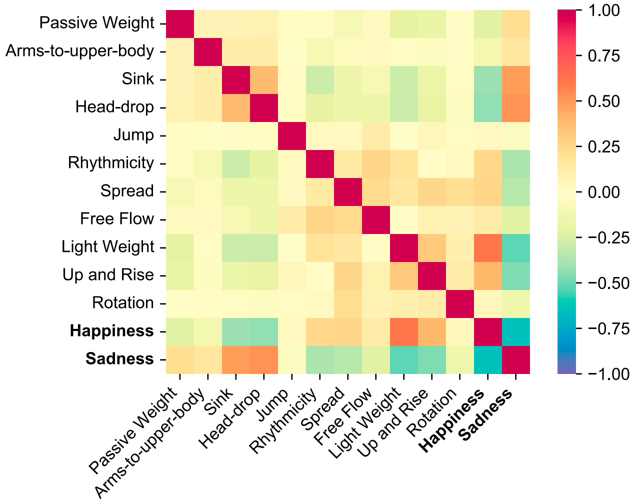

Figure 3. Correlation among LMA elements and emotions

Emotion categories are written in bold. This confirms the association between the selected LMA elements and the emotions of happiness and sadness.

All presented methods can be trained from scratch or pretrained using various existing datasets. Pretraining refers to the process of initially training a model on one dataset before fine-tuning it on the target dataset, in this case, BoME. In Table 2, we leveraged the image classification datasets ImageNet-1K/ImageNet-22K[36] and the video recognition dataset Kinetics-400[37] for pretraining purposes. For other aspects, such as the testing strategy, we followed the implementation guidelines provided by the MMaction2 codebase.[35]

Our analysis of Table 2 yields several key insights. Firstly, pretraining significantly enhances the performances of all four algorithms. Specifically, pretraining with ImageNet leads to a notable improvement compared with training from scratch, with gains of 5.72 mAP(%) and 6.48 mRA(%) for TSN, and 3.57 mAP(%) and 2.99 mRA(%) for V-Swin. Pretraining on Kinetics-400 produces even more substantial improvements, with increases of 8.32, 10.07, 14.09, and 5.87 mAP(%) for TSN, SlowFast, V-Swin, and PoseC3D, respectively. This is expected, as the Kinetics-400 dataset is specifically designed for human activity classification, and some human characteristic features can be transferred to the LMA estimation task.

Moreover, our results suggest that the sample rate at which the input clip is extracted into frames may impact performance. A higher density of frame samples within a clip may allow for more information to be extracted, but may also hinder the model's ability to analyze such densely packed frames, leading to a decrease in performance. TSN achieves the best performance when the clip is split into 16 sub-clips. SlowFast and V-Swin exhibit worse performance with denser sampling rates than the default rate. PoseC3D performs optimally with a sampling rate of 72 frames per clip, the densest among the four algorithms. This may be because PoseC3D uses skeleton coordinates as input, which may be easier for the neural network to interpret compared with images.

Finally, our results show that V-Swin achieves the best performance (53.67 mAP(%) and 72.82 mRA(%)) among the four algorithms, with 32 samples per clip and pretraining on Kinetics-400. Despite using only skeleton input, PoseC3D attains competitive mAP performance compared with TSN and SlowFast.

Based on the mAP values, we have selected the best-performing model for each algorithm. Some qualitative examples of LMA element estimation by different models, along with the ground truth (i.e., the labels provided by the CMA), are shown in Figure 5. We also conducted a breakdown analysis. Figure 6 presents the precision-recall curves for various models across all the LMA elements. Notably, V-Swin outperforms the other algorithms on the light weight and rotation elements. PoseC3D performs particularly well on the passive weight and arms-to-upper-body elements, but poorly on the spread and rotation elements.

LMA enhances bodily expressed emotion understanding

In this subsection, we aim to achieve the ultimate goal of enhancing BEEU by integrating LMA element labels. As aforementioned, several psychological studies have demonstrated a strong correspondence between the 11 LMA elements and emotion categories sadness and happiness.[24–26] Our previous statistical analysis has confirmed this finding in the BoME dataset. Furthermore, we have shown that deep neural networks can learn an effective body movement representation from BoME. The optimal performance for LMA element estimation on BoME is 53.67 mAP(%) and 72.72 mRA(%), which is significantly higher than the best results achieved in BEEU (19.30 mAP(%) and 66.94 mRA(%)) on the bodily expression benchmark BoLD. This is likely due to the fact that LMA elements have a more objective definition than emotion categories, as the presence of LMA elements in a human clip depends solely on the body movement, whereas emotion labels may also be influenced by the annotators' emotional state. In summary, emotion and LMA element labels are related, and LMA element labels are easier for deep neural networks to learn. Thus, incorporating the human movement features learned from BoME into BEEU presents a promising approach.

To achieve our goal of improving BEEU, we need to train and test on the BEEU benchmark dataset BoLD, using the BoME

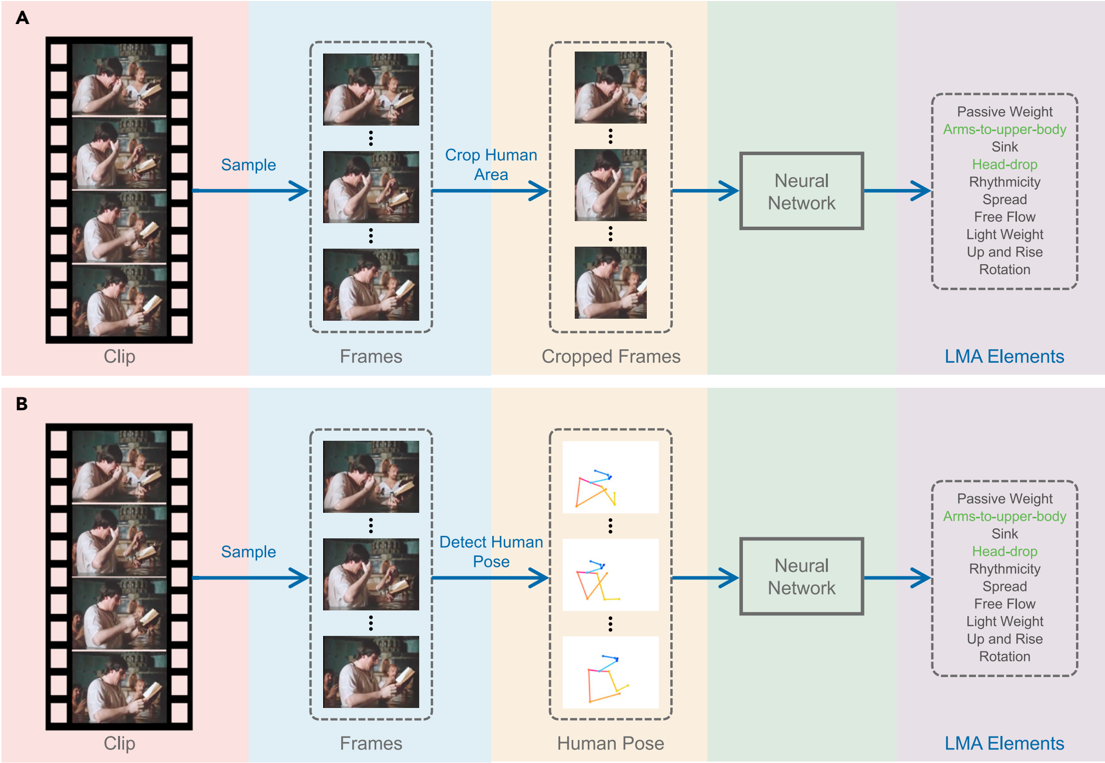

Figure 4. RGB-based and skeleton-based pipelines for estimating the LMA elements

(A) The RGB-based pipeline extracts frames from the input clip, crops the target human, and feeds the resultant frames into a neural network.

(B) The Skeleton-based pipeline leverages the 2D/3D human pose extracted from the frames as the input for a neural network.

This figure incorporates frames from the film "Wagner" (1983, directed by Tony Palmer).

dataset as an additional training source. In this set of experiments, we jointly trained on the BoLD training set and the entire BoME dataset, and then evaluated the model on the BoLD validation and test sets. It is worth noting that BEEU on BoLD, like LMA estimation, involves multi-label binary classification tasks. We have also adopted mAP and mRA as evaluation metrics.

Figure 7 illustrates the proposed method involving the creation of a dual-branch, dual-task neural network, named MANet. We adopt the same input processing technique as SlowFast and V-Swin from the previous subsection. By sampling 48 frames from the input clip and subsequently employing OpenPose[32] to detect the human region within these frames, we crop and resize the area to 224×224 as input, resulting in an input shape of 48 × 224 × 224. The processed frames then fed into the neural network.

We have implemented two essential design elements in the neural network to enable LMA annotations to support BEEU. First, as shown in Figure 7, we designed a dual-branch network structure that allowed the model to produce LMA and emotion predictions concurrently. We employed Swin blocks (i.e., architecture building blocks in V-Swin[28]) to construct the MANet's backbone, followed by the emotion branch and the LMA branch. The LMA branch extracts LMA features from the backbone's output and utilizes a linear classifier to generate the LMA output. Second, we incorporated a fusion operation by combining the LMA branch features with the emotion features, enabling the emotion predictions to be informed by human movement features. In the emotion branch, the fused features are fed into a linear classifier to yield the emotion output. We provide more details of the model structure in the experimental procedures.

We employed various loss functions to supervise the training of MANet. As both emotion and LMA predictions involve multi-label binary classification tasks, we utilized the multi-label binary cross-entropy loss to compute emotion loss and LMA loss by comparing their respective outputs with ground truth labels. Furthermore, we introduced the bridge loss to create a connection between LMA and emotion prediction based on the relationship between LMA and specific emotion categories (i.e., sadness and happiness). Importantly, we used a threshold ϵ in bridge loss to control the extent of LMA prediction supervision over emotion prediction.

Moreover, we utilized a weakly supervised training approach to enable joint training despite the fact that some BoLD samples lack LMA labels and some BoME samples are missing emotion labels. Comprehensive information on the loss function design and training procedure can be found in the experimental procedures.

Table 3 presents the results of the ablation study. The first set of experiments evaluates the impact of the model structure on performance. The method without the dual-branch and fusion components refers to training emotion labels using only the original V-Swin architecture. In this case, the performance is 19.97 mAP(%) and 67.16 mRA(%) on the BoLD validation set.

Table 2. Benchmarking the BoME dataset with four deep learning-based methods

Method	Type	Pretrain	mAP (%)	mRA (%)	Samples	FLOPs ($\times 10^9$)	Param. ($\times 10^6$)
TSN	RGB based	Scratch	40.71	60.42	8 × 1	7.3	23.5
TSN	RGB based	ImageNet-1K	46.43	66.90	8 × 1	7.3	23.5
TSN	RGB based	Kinetics-400	49.03	68.58	8 × 1	7.3	23.5
TSN	RGB based	Kinetics-400	**51.02**	69.91	16 × 1	7.3	23.5
TSN	RGB based	Kinetics-400	50.58	**70.35**	24 × 1	7.3	23.5
SlowFast	RGB based	Scratch	39.20	59.28	1 × 32	174	62.0
SlowFast	RGB based	Kinetics-400	**49.27**	**69.23**	1 × 32	174	62.0
SlowFast	RGB based	Kinetics-400	48.62	68.19	1 × 48	260	62.0
SlowFast	RGB based	Kinetics-400	47.70	68.12	1 × 64	347	62.0
V-Swin	RGB based	Scratch	39.58	59.33	1 × 32	282	88.1
V-Swin	RGB based	ImageNet-1K	43.15	62.32	1 × 32	282	88.1
V-Swin	RGB based	ImageNet-21K	48.88	66.49	1 × 32	282	88.1
V-Swin	RGB based	Kinetics-400	**53.67**	**72.82**	1 × 32	282	88.1
V-Swin	RGB based	Kinetics-400	50.78	69.55	1 × 48	423	88.1
V-Swin	RGB based	Kinetics-400	46.79	64.42	1 × 64	564	88.1
PoseC3D	skeleton based	Scratch	38.88	58.45	1 × 48	15	3.0
PoseC3D	skeleton based	Kinetics-400	44.75	61.75	1 × 48	15	3.0
PoseC3D	skeleton based	Kinetics-400	**50.30**	64.72	1 × 72	22	3.0
PoseC3D	skeleton based	Kinetics-400	46.93	**64.93**	1 × 96	29	3.0

"Samples" means number of sub-clip × number of frames per sub-clip in training when sampling one input clip. "FLOPs" represent the computational complexity of a neural network. "Param." determines the network's size and capacity to learn. The numbers highlighted in bold represent the best performance in each method.

Incorporating the dual-branch structure, but omitting fusion, does not significantly improve performance compared with the original V-Swin. However, the fusion operation leads to a 0.46 mAP(%) and 0.60 mRA(%) increase over the original V-Swin. This suggests that multi-task training and feature fusion are both necessary for improving BEEU. The effectiveness of the bridge loss is also analyzed. As detailed in the experimental procedures, the initial version of the bridge loss does not incorporate the threshold ϵ, and its performance does not differ significantly from the model without the loss. However, by adding ϵ and setting it to 0.9, the bridge loss leads to a significant improvement of 0.82 mAP(%) and 0.56 mRA(%). Thus, the final MANet model consists of the bridge loss, dual-branch structure, and fusion operation, yielding an overall mAP increase of 6.4% (from 19.97 to 21.25). Qualitative examples of bodily expression estimation using the final model and two baselines are shown in Figure 8.

The central idea of the bridge loss is to use LMA prediction to supervise the prediction of sadness and happiness. To further investigate the impact of the bridge loss on these two emotion categories, we present precision-recall curves for sadness and happiness in Figure 9 for the final model and two baselines. These results show that the bridge loss leads to a significant improvement in sadness, with an increase of 8.75 AP(%) and 9.06 AP(%) over baseline-1 and baseline-2, respectively. There is also a notable improvement in the happiness category. In addition, Table S1 provides an analysis across all emotion categories.

Table 4 presents a comparison of MANet's performance with that of previous state-of-the-art methods on the BoLD validation and test sets. Our study re-implemented the state-of-the-art emotion work by Beyan et al.[20] We achieved this using the public code they provided, applied specifically to the BoLD dataset. We collected performance data for other competitive models from their papers or from the ARBEE work.[6] Results from the table show that MANet outperforms the approach by Pikoulis et al.[38] by 1.95 mAP(%) and 1.38 mRA(%) on the BoLD validation set. On the test set, the single model of MANet delivers comparable performance with the model ensembles of Pikoulis et al. Employing the same ensemble strategy, the mAP of MANet surpasses the work of Pikoulis et al.[38] by 5.6%. The superior performance of MANet is attributed to its use of BoME as an additional source of training data, despite its smaller size (approximately one-sixth of the BoLD training set). Compared with Beyan et al.,[20] our method exhibits substantially better performance. The difference in performance may arise from two factors. First, our study focuses on in-the-wild data, whereas Beyan et al. concentrates on lab-collected data, leading to a domain gap. Second, Beyan et al. relied on 3D motion capture data, which is not available for the BoLD dataset. Instead, we used OpenPose[32] to extract 2D pose data as input, which may have reduced the performance of Beyan et al. Despite these differences, Beyan et al.'s state-of-the-art emotion work still outperforms some other skeleton-based methods.

DISCUSSION

In this study, we present BoME, an innovative dataset grounded in LMA for human motor elements. We showcase the effectiveness of deep neural networks in capturing human movement

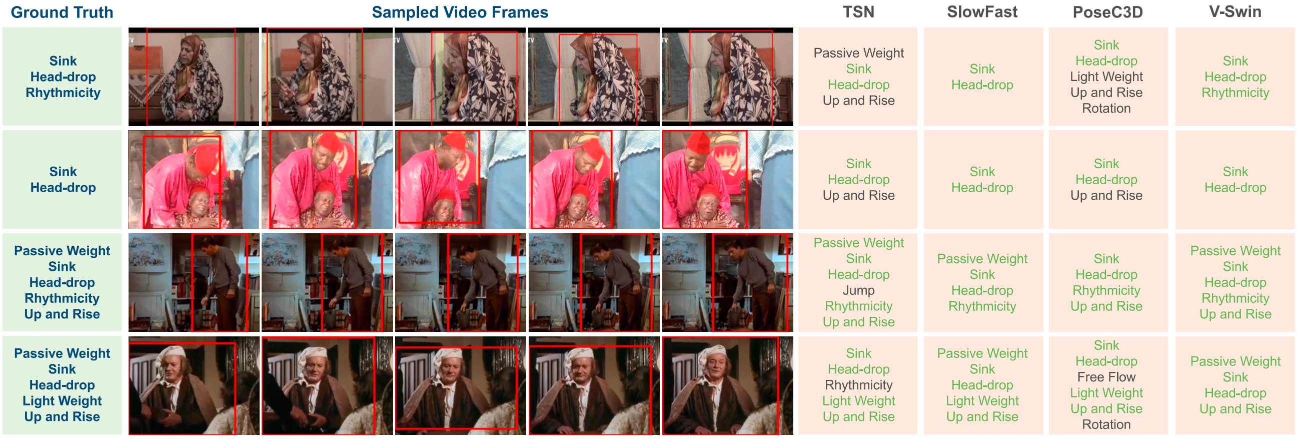

Figure 5. Example LMA element estimation results on the BoME dataset

Five frames sampled from each clip are shown. The predicted LMA elements that are also in the ground truth list are shown in green. The figure incorporates frames from the films, listed from top to bottom, "Por mi Hermana" (2013), "Town in Danger - Nigerian Nollywood Movie" (2003), "Charly" (1968, directed by Ralph Nelson), and "The Black Velvet Gown" (1991, directed by Norman Stone), respectively.

representation through the utilization of this dataset. Furthermore, we propose MANet, a cutting-edge dual-branch model designed for the understanding of bodily expressed emotions. This model harnesses the supervisory information provided by BoME, employing a specialized model architecture, a custom loss function, and a weakly supervised training strategy. As a result, MANet surpasses existing approaches in the domain of BEEU.

This study employs 11 distinct LMA elements known to be related to sadness and happiness to enhance BEEU. With the LMA system encompassing over 100 elements, there is considerable potential for additional elements to contribute to emotion recognition. To build upon this work, we suggest two main avenues for future endeavors. First, it is recommended to expand the dataset and enrich it with more annotations, incorporating a broader range of LMA element labels and emotion labels. Second, we anticipate that further research in the fields of psychology and affective computing could reveal valuable insights into the relationships between LMA elements and emotions. Such advancements would ultimately enhance BEEU research and facilitate a deeper understanding of human emotions as expressed through movement.

This work has established that LMA contributes significantly to the task of BEEU. LMA could potentially enhance other computer vision tasks as well, such as general human action recognition. It is evident that certain LMA elements are associated with specific human actions. For instance, in sports activities like tennis, players swing their rackets, and swimmers exhibit distinct strokes. The LMA system utilizes various labels to describe these actions. Similarly, in human social activities, certain actions, such as shaking hands, are characteristic, and the LMA system can assist in recognizing them. However, as mentioned earlier, this would necessitate additional LMA element annotation, as the current 11 elements are not sufficient. In the future, we may consider expanding the LMA annotation labels to facilitate the analysis of a broader range of human activities.

Our exploration of LMA and emotion recognition holds significant potential for practical applications across various domains, particularly those where explanation and understanding are crucial or preferred. One such area is the medical field, particularly in the care of mental health patients. By monitoring patients' body movement patterns, healthcare professionals can be alerted about a need to directly observe their emotional states and an explanation can be given. This approach could improve efficiency in patient care. Another notable application is in robotics and human-computer interaction. Empowering robots with the ability to recognize human emotions through body movements, and to adapt their models based on repeated observations of an individual's movements, paves the way for more informed interaction decisions grounded in the individuals' emotional states. With LMA motor element recognition, a robot can incorporate different types of movement into its decision-making process due to the diverse emotional significance each carries. This advancement fosters a more personalized, natural, and empathetic human-robot interaction experience. A recent review article discusses additional example applications of improved BEEU.[3]

In summary, incorporating LMA elements has effectively enhanced BEEU and shows promise for further advancements in the future.

EXPERIMENTAL PROCEDURES

Resource availability

Lead contact

Request for information and resources used in this article should be addressed to Dr. James Wang (jwang@ist.psu.edu).

Materials availability

This study did not generate new unique reagents.

Data and code availability

The BoME dataset, along with the model training and evaluation code, are available at Mendeley Data: https://doi.org/10.17632/gbhpdkf8pg.1. They are also available at GitHub: https://github.com/ChenyanWu/BoME. All the code used in the experiments was implemented with PyTorch. We developed the code based on the open-source codebase MMaction2. Part of the code includes the BoLD dataset as the training set. BoLD is publicly available at URL: http://cydar.ist.psu.edu/emotionchallenge.

CellPress
OPEN ACCESS

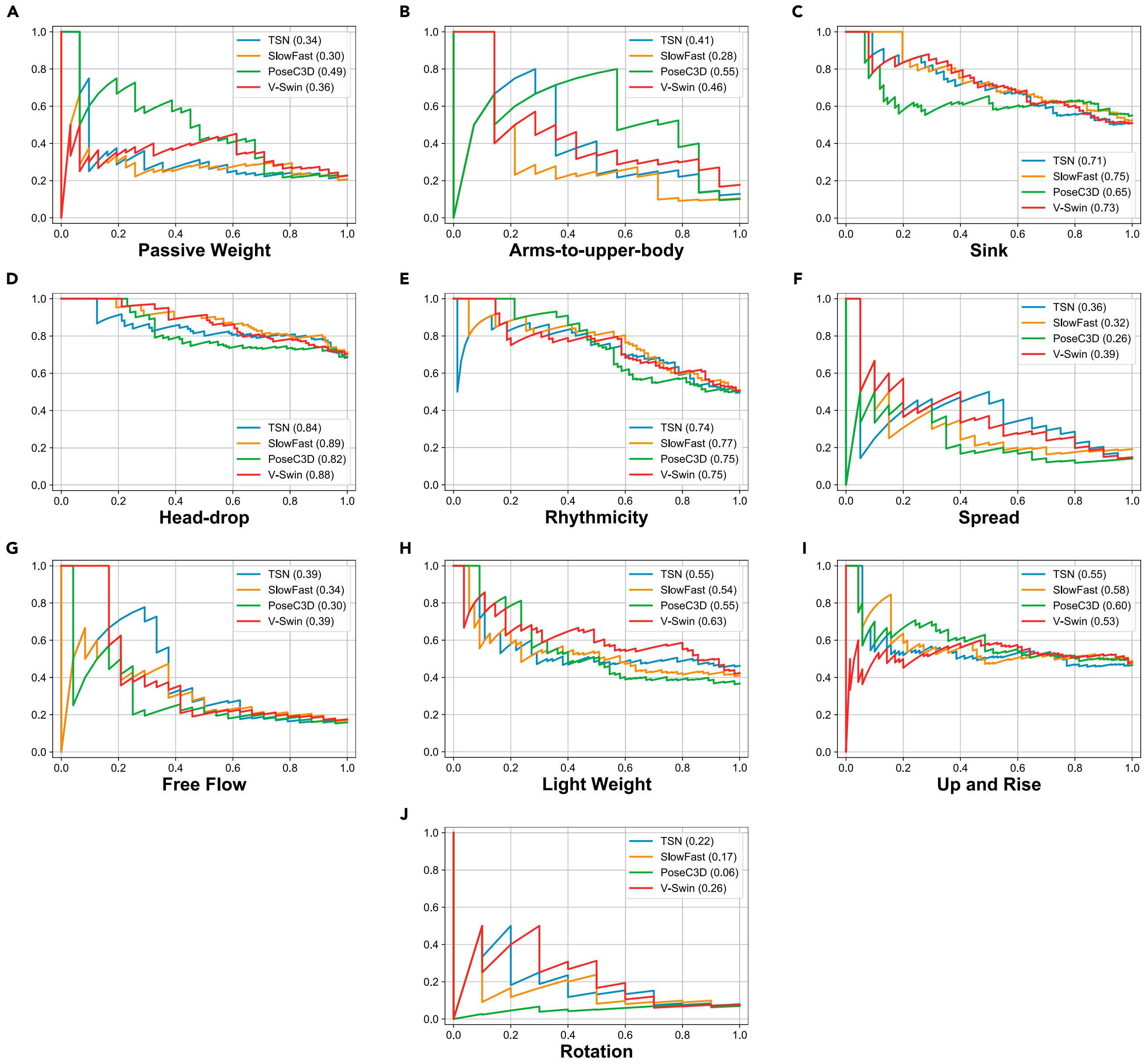

Figure 6. Precision-recall curves for various models on 10 LMA elements

The x axis represents the recall and the y axis represents the precision. The AP (average precision) score of the corresponding model is indicated in parentheses after the model name in the legend.

Selecting motor element labels

To characterize motor elements, we adopted the LMA, the most extensively developed system for encoding human movement. Rudolf von Laban (1879–1958), a renowned dance artist, choreographer, and movement theorist, spearheaded the development of LMA in the early 20th century to analyze and record body movements in dance, theater, education, and industry. The LMA system comprises over one hundred motor elements, organized into four main categories. The body category lists moving body parts (such as the head and arms) and some basic actions (such as jumping and walking). The space category represents the body's spatial direction when moving, including vertical (up, down), sagittal (forward, backward), and horizontal (right side/left side). The shape category describes how the body changes its shape, including whether it encloses or spreads, rises or sinks. The effort category specifies the mover's inner attitude toward the movement and is expressed in the quality of the movement. It is comprised of four factors: weight, space, time, and flow. Weight-effort refers to the amount of force applied by the mover, with a spectrum ranging from strong (applying high force) to light (applying weak force). Space-effort ranges from direct to indirect, indicating whether the mover moves directly toward a target in space or indirectly. Time-effort ranges from sudden to sustain, denoting the movement's acceleration. Flow-effort ranges from bound to free flow, expressing the level of control exerted over the movement. LMA also includes the phrasing category, which describes how the motor elements change over time.

Several studies have demonstrated that certain LMA elements are strongly associated with emotions, particularly sadness and happiness. Shafir et al.[24] found that specific LMA elements, when present in a movement, can elicit four fundamental emotions, including sadness and happiness, among others. Melzer et al.[25] identified LMA elements that allow movements to be classified

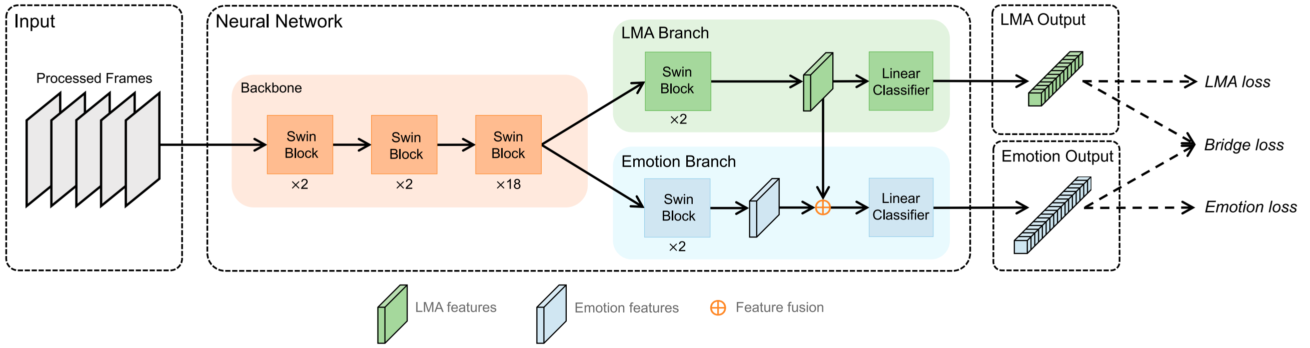

Figure 7. The framework of the proposed MANet

MANet takes processed frames from video clips as input. The network is composed of a backbone and two distinctive branches—the LMA and emotion branches. Both branches ingest the output of the backbone, extract relevant features, and subsequently yield separate LMA and emotion outputs. Of note, within the emotion branch, a fusion operation takes place, integrating the emotion features with the LMA features. The network training is facilitated by the application of three loss functions: LMA, bridge, and emotion.

as expressing one of these four fundamental emotions. The association between happiness and certain LMA elements was also validated by van Geest et al.[26] Furthermore, another experiment by the Shafir group, conducted by Gilor for her Master's thesis, studied LMA elements used for expressing sadness and happiness. These studies provide compelling evidence that certain LMA elements can evoke or be recognized as conveying sadness and happiness. Our work builds upon these psychological findings and, following Shafir et al.[24] and Melzer et al.,[25] we selected 11 LMA elements associated with sadness and happiness (see Table 1) as the labels for the BoME dataset. All experiments presented in this paper are based on these motor elements.

The creation of the BoME dataset

To create the BoME dataset, we followed the same process as the BoLD dataset by using movies from the AVA dataset[23] as our data source. This has two advantages. First, real-world video recordings often have limited body movements, but movies provide a rich variety of visual features. Second, we can match video clips from the BoLD dataset, which have emotion category labels, for joint training and emotion modeling.

The films of the AVA dataset are sourced from YouTube, and all associated copyrights are retained by the original content creators. In addition, the Common Visual Data Foundation (CVDF) hosts these videos.[41] During our research, we retrieved the videos from the CVDF using their GitHub repository.[41] The CVDF offers a stable and reliable platform for researchers. In adherence to copyright regulations and the procedures established by the AVA dataset, our BoME dataset includes only the YouTube IDs of the films. This allows users to access the corresponding videos from either YouTube or the CVDF using these identifiers. It should be noted that, while the videos in this dataset are intended to comply with YouTube's guidelines, which strictly prohibit explicit content such as violence and nudity, users should remain aware of the potential presence of harmful or sensitive material. Despite the diligent oversight from both YouTube and our team, occasional oversights may occur, resulting in the inclusion of such content. Users are advised to exercise discretion while accessing these videos.

To segment the long movies from the AVA dataset into clips, we employed the kernel temporal segmentation approach,[42] consistent with BoLD. The selection of clips was carried out by the LMA annotator, adhering to certain criteria: (1) the human subject in the clip must display clearly discernable emotions, specifically sadness or happiness, as our study is focused on the 11 motor elements associated with these two emotions according to previous research. (2) The clip should be brief, with fewer than 300 frames in total, since longer clips may contain expressions of multiple emotions, making it difficult to attribute each LMA label to the correct emotion. (3) We excluded clips in which the human subject did not display any movement. After careful screening, we ultimately chose 1,600 clips to form the BoME dataset. In the BoME dataset, we supply the initial and terminal frame numbers for each clip, enabling users to precisely locate these segments within the context of the original film.

Because each video may contain multiple people, we need to identify which person to annotate. We adopted the method proposed by Luo et al.[6] for human identification. Specifically, we leveraged the pose estimation network OpenPose[32] to extract the coordinates of the human joints, which allowed us to determine a bounding box around the person's body. By implementing a tracker on this bounding box, we assigned a unique identification number to the same person in all frames of the video clip, enabling consistent annotation across the entire duration of the clip.

We enlisted the assistance of an LMA expert to provide annotations for the study. This expert is a member of a team that has received specialized training in LMA coding for scientific research and has already coded numerous hours of movements for previous quantitative studies using the same standard annotation pipeline as in our research.

The annotator was instructed to ensure that the sound in the clips was turned off during annotation to prevent any influence from auditory cues, such as tone of voice or background music, which could impact the perceived emotion. Instead, the annotator was to focus solely on the observed movements. Each clip was watched multiple times by the annotator to code all 11 variables, which are the 11 motor elements that have been linked to motor expressions of sadness and happiness in previous psychological studies. The annotator coded some of the variables during each viewing and repeated the process until all variables were coded. The annotator then watched the clip one last time to verify the accuracy of the annotations. If the LMA expert was uncertain about the correct coding, she was instructed to move and match what she saw in the clip with her own body movement and even intensify it when necessary until it became clear which motor elements constituted the movement.

The LMA expert used a standardized and consistent rating scale of 0–4 to code each motor element, taking into account both its duration (i.e., the

Table 3. Ablation on the architecture and bridge loss

Dual-branch	Fusion	Bridge loss	mAP (%)	mRA (%)
–	–	–	19.97	67.16
✓	–	–	19.94	67.34
✓	✓	–	20.43	67.76
✓	✓	w/o ε	20.42	66.88
✓	✓	$\varepsilon = 0.7$	19.78	67.44
✓	✓	$\varepsilon = 0.8$	20.55	67.43
✓	✓	$\varepsilon = 0.9$	21.25	68.32
✓	✓	$\varepsilon = 0.99$	20.67	67.57

Evaluation is done on the BoLD validation set.

CellPress
OPEN ACCESS

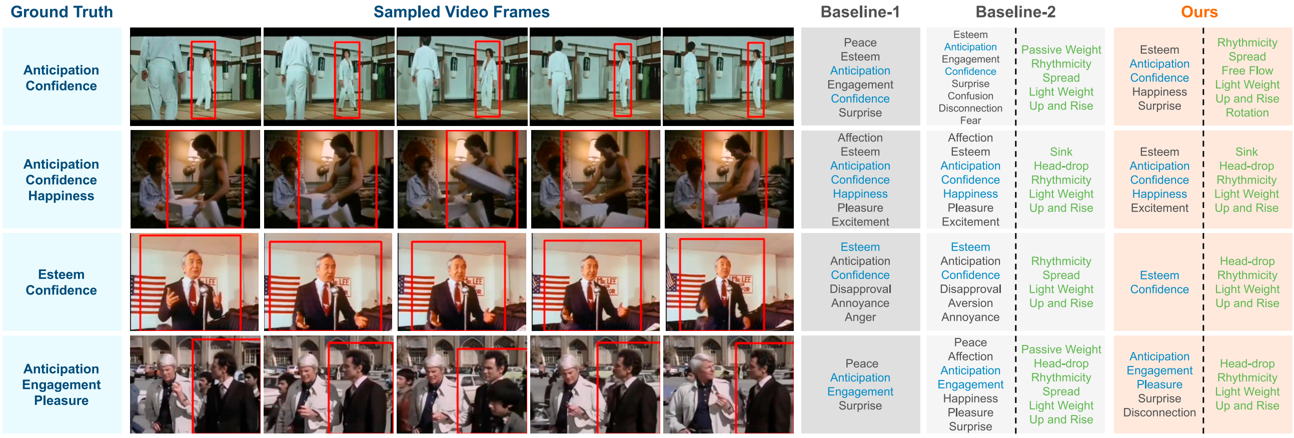

Figure 8. Example bodily expressed emotion understanding results on the BoLD validation set

Categorical emotion labels are predicted based on a video clip of a person. Five frames sampled from each clip are shown. The predicted emotion labels that are also in the ground truth list are shown in blue. Baseline-1 is the original V-Swin without dual-branch and fusion. Baseline-2 is the MANet model without the bridge loss. Ours is the final model of MANet. We also present the predicted LMA elements in green. Baseline-1 does not provide LMA predictions because it only outputs emotion prediction. The figure incorporates frames from the films, listed from top to bottom, "Return of the Tiger" (1978, directed by Jimmy Shaw), "Ragin' Cajun" (1990, directed by William Byron Hillman), "Eye of the Stranger" (1993, directed by David Heavener), and "Teheran Incident" (1979, directed by Leslie H. Martinson).

percentage of clip duration during which the motor element was observed) and intensity. To determine the duration score, the following criteria were used.

- 0: the motor element was not observed in the clip.
- 1: the motor element was rarely observed, appearing for up to a quarter of the clip duration.
- 2: the motor element was observed a few times, appearing for up to half of the clip duration.
- 3: the motor element was often observed, appearing for up to three-quarters of the clip duration.
- 4: the motor element was observed for most or all of the clip duration.

If the intensity of the motor element was low, 1 was subtracted from the duration score. Conversely, if the intensity was high, 1 was added to the duration score. However, the maximum score could not exceed 4.

Model structure of MANet

As illustrated in Figure 7, the network consists of a single backbone followed by two branches: the emotion branch and the LMA branch. The backbone is responsible for extracting image features from the input frames, and it strictly adheres to the first three stages of V-Swin.[28] Each stage comprises multiple 3D Swin Transformer blocks, the structure of which is detailed in the V-Swin paper.[28] We employed the base setting of V-Swin, which includes 2, 2, and 18 blocks in the first three stages, respectively. The LMA branch utilizes two 3D Swin Transformer blocks to process the output from the backbone and obtain the LMA features. Subsequently, a linear classifier within the LMA branch generates the LMA prediction. Similarly, the emotion branch employs two 3D Swin Transformer blocks to extract the emotion features. Following this, the emotion features and the LMA features are added through a feature fusion operation. The fused features are then input into a linear classifier, which ultimately produces the emotion output.

Loss functions of MANet

As depicted in Figure 7, MANet is trained by optimizing three loss functions: emotion loss, LMA loss, and bridge loss.

The emotion output is represented as a vector $y = [y_0, y_1, \ldots, y_N]$, with N denoting the number of emotion categories (26 for BoLD). Similarly, the LMA output is expressed as $z = [z_0, z_1, \ldots, z_M]$, with M representing the number of LMA elements (10 for BoME). We apply the sigmoid function to y_i, indicated as $\sigma(y_i)$, to determine the probability that the input sample encompasses the ith label. The same is applied to z_j.

Let the ground truth emotion and LMA labels be $\hat{y} = [\hat{y}_0, \hat{y}_1, \ldots, \hat{y}_N]$ and $\hat{z} = [\hat{z}_0, \hat{z}_1, \ldots, \hat{z}_N]$. For any $\hat{y}_i$, the value is either 1 or 0, indicating whether the ith label is true or false for the sample. The same is applied to $\hat{z}_j$.

We calculated the emotion loss and LMA loss by computing the cross-entropy between the ground truth and output predictions as follows:

$$\mathcal{L}_{\text{Emotion}} = -\frac{1}{N}\sum_{i=1}^{N} \hat{y}_i \ln \sigma(y_i) + (1 - \hat{y}_i)\ln(1 - \sigma(y_i)),$$

$$\mathcal{L}_{\text{LMA}} = -\frac{1}{M}\sum_{j=1}^{M} \hat{z}_j \ln \sigma(z_j) + (1 - \hat{z}_j)\ln(1 - \sigma(z_j)) .$$

Previously, we established that the first four LMA elements were associated with sadness, while the remaining elements were associated with happiness.

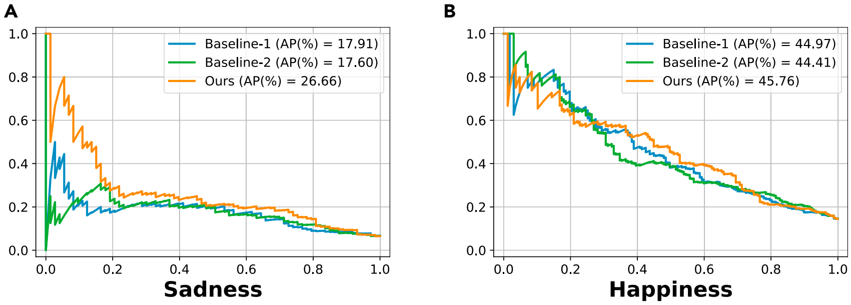

Figure 9. Precision-recall curves for sadness and happiness on the BoLD validation set

Models of baseline-1, baseline-2, and ours are identical to those in Figure 8.

Table 4. Comparison with the state of the art on the BoLD validation and test set

Set	Method	Year	mAP (%)	mRA (%)
Validation	TSN[29]	2018	18.55	64.27
	Filntisis et al.[8]	2020	16.56	62.66
	Pikoulis et al.[38]	2021	19.30	66.94
	Beyan et al.[20,b]	2021	15.86	62.63
	MANet	2023	**21.25**	**68.32**
Test	I3D[39]	2017	15.37	61.24
	TSN[29]	2018	17.02	62.70
	ST-GCN[40,b]	2018	12.63	55.96
	Filntisis et al.[8]	2020	17.96	64.16
	random forest[6,b]	2020	13.59	57.71
	Pikoulis et al.[38,a]	2021	21.87	68.29
	Beyan et al.[20,b]	2021	16.73	62.17
	MANet	2023	22.11	67.69
	MANet[a]	2023	**23.09**	**69.23**

The numbers highlighted in bold represent the best performance in the validation or test set.
[a]Represents model ensembles.
[b]Represents skeleton-based method.

Utilizing this information, we developed the bridge loss to guide the prediction of sadness and happiness. Specifically, for $z = [z_0, z_1, \ldots, z_M]$, we selected the maximum predictions among the sadness- and happiness-related LMA elements, denoted as max $\{z_i\}_{i=1}^{4}$ and max $\{z_i\}_{i=5}^{M}$, respectively. By calculating the softmax of two values, we obtained the probabilities for sadness and happiness. Formally, we have,

$$p_{\text{sadness}} = \frac{e^{\max \{z_i\}_{i=1}^{4}}}{e^{\max \{z_i\}_{i=1}^{4}} + e^{\max \{z_i\}_{i=5}^{M}}},$$

$$p_{\text{happiness}} = \frac{e^{\max \{z_i\}_{i=5}^{M}}}{e^{\max \{z_i\}_{i=1}^{4}} + e^{\max \{z_i\}_{i=5}^{M}}}.$$

The variables $p_{\text{happiness}}$ and p_{sadness} are considered as probabilities from the perspective of LMA predictions. Let the sth and h^{th} elements of vector y denote sadness and happiness, respectively. By applying the softmax function to y_h and y_s, we derived the probabilities of happiness and sadness from the emotion output, represented as $e^{y_h}/(e^{y_h} + e^{y_s})$ and $e^{y_s}/(e^{y_h} + e^{y_s})$, respectively. Subsequently, we leveraged $p_{\text{happiness}}$ and p_{sadness} to supervise $e^{y_h}/(e^{y_h} + e^{y_s})$ and $e^{y_s}/(e^{y_h} + e^{y_s})$ through the implementation of the soft cross-entropy loss:

$$\mathcal{L}_{\text{Bridge}} = - p_{\text{happiness}} \ln \frac{e^{y_h}}{e^{y_h} + e^{y_s}} - p_{\text{sadness}} \ln \frac{e^{y_s}}{e^{y_h} + e^{y_s}}.$$

Occasionally, the happiness-sadness probability from the LMA branch may not be accurate, hindering its ability to supervise y_h and y_s. To address this issue, we introduced a threshold ϵ. Only when $p_{\text{happiness}}$ or p_{sadness} exceeded ϵ would we compute the cross-entropy loss. Formally, this can be represented as:

$$\mathcal{L}_{\text{Bridge}} = -1(p_{\text{happiness}} > \epsilon)\ln \frac{e^{y_h}}{e^{y_h} + e^{y_s}} - 1(p_{\text{sadness}} > \epsilon)\ln \frac{e^{y_s}}{e^{y_h} + e^{y_s}},$$

where $1(P)$ is the indicator function, equating to 1 if the condition P is true and 0 otherwise. An ablation study was conducted to evaluate the performance of different $\mathcal{L}_{\text{Bridge}}$ values, as shown in Table 3. The results suggest that the ϵ-controlled loss function leads to improved performance, with $\epsilon = 0.9$ achieving the best results.

Weakly supervised training for MANet

We have utilized both the BoME and BoLD datasets for the joint training of MANet to recognize emotion and LMA labels. These datasets share a common subset of 705 clip samples. The rest of the BoME samples are exclusive to LMA labels, whereas the remaining samples in the BoLD set contain only emotion labels. Consequently, drawing inspiration from the work of Wu et al.,[43] we adopted a weakly supervised training methodology, enabling us to effectively leverage data that lack either emotion or LMA labels.

In particular, we employed the coefficient μ_{Emotion}, set to either 0 or 1, to indicate the presence or absence of an emotion label in a sample. Likewise, the coefficient μ_{LMA} was used for the LMA label. During training, all datasets were combined and shuffled together. We utilized the coefficients λ_1 and λ_2 to balance the three loss components. The total loss was calculated as follows:

$$\mathcal{L} = \mu_{\text{Emotion}}\mathcal{L}_{\text{Emotion}} + \lambda_1 \mu_{\text{LMA}}\mathcal{L}_{\text{LMA}} + \lambda_2 \mathcal{L}_{\text{Bridge}},$$

in practice, we set $\lambda_1 = 0.25$ and $\lambda_2 = 0.1$.

Throughout the training process, the network was trained for 50 epochs, with data augmentation techniques such as flipping and scaling applied to both the BoME and BoLD datasets. The learning rate was set at 5e−3, and the optimization algorithm employed was SGD. Two NVIDIA Tesla V100 GPUs were used to conduct a single experiment, which took approximately 8 h to complete.

SUPPLEMENTAL INFORMATION

Supplemental information can be found online at https://doi.org/10.1016/j.patter.2023.100816.

ACKNOWLEDGMENTS

The work was supported by generous gifts from the Amazon Research Awards program to J.Z.W. and by the National Science Foundation (NSF) under grant nos. 2234195 and 2234197. This work used cluster computers at the Pittsburgh Supercomputer Center through an allocation from the Advanced Cyberinfrastructure Coordination Ecosystem: Services & Support (ACCESS) program, which is supported by NSF grants nos. 2138259, 2138286, 2138307, 2137603, and 2138296. This work also used the Extreme Science and Engineering Discovery Environment, which was supported by NSF grant no. 1548562. We are grateful to Laura Schandelmeier for providing the LMA annotations for BoME and to Yelin Kim and Adam Fineberg for their support and encouragement. We would also like to express our appreciation to the anonymous reviewers for their constructive feedback.

Some movie frames are incorporated in figures for illustrative purposes and to support the conceptual discussions in this paper. All copyrights remain with their respective owners. The authors acknowledge and appreciate the work of the movie creators.

AUTHOR CONTRIBUTIONS

Conceptualization, C.W. and J.Z.W.; methodology, software, validation, investigation, and writing – original draft, C.W.; formal analysis, C.W. and D.D.; data curation, C.W., T.S., and R.T.; writing – review & editing, C.W., T.S., R.T., and J.Z.W.; visualization, C.W. and D.D.; resources, supervision, project administration, and funding acquisition, J.Z.W.

DECLARATION OF INTERESTS

J.Z.W. is a named inventor on US patent 11,636,368, "Probabilistic multigraph modeling for improving the quality of crowdsourced affective data," and is a named inventor on the US patent application titled "Systems and methods for automated recognition of bodily expression of emotion." J.Z.W. and C.W. are named inventors on the US provisional patent application titled "Systems and methods for learning emotion representations from verbal and nonverbal communication." They are all assigned to The Penn State Research Foundation. These interests do not influence the integrity of the research, and all efforts have been made to ensure that the research was conducted and presented in an unbiased manner.

CellPress
OPEN ACCESS

DECLARATION OF AI AND AI-ASSISTED TECHNOLOGIES IN THE WRITING PROCESS

During the preparation of this work, the authors used ChatGPT in order to improve the readability and language of the work. After using this tool, the authors reviewed and edited the content as needed and take full responsibility for the content of the publication.

Received: December 27, 2022
Revised: May 21, 2023
Accepted: July 13, 2023
Published: August 22, 2023

REFERENCES

1. Marcos-Pablos, S., and García-Peñalvo, F.J. (2021). Emotional intelligence in robotics: A scoping review. In Proc. Int. Conf. Disruptive Technologies, Tech Ethics and Artificial Intelligence (Springer), pp. 66–75. https://doi.org/10.1007/978-3-030-87687-6_7.
2. Cowie, R., Douglas-Cowie, E., Tsapatsoulis, N., Votsis, G., Kollias, S., Fellenz, W., and Taylor, J.G. (2001). Emotion recognition in human-computer interaction. IEEE Signal Process. Mag. *18*, 32–80. https://doi.org/10.1109/79.911197.
3. Wang, J.Z., Zhao, S., Wu, C., Adams, R.B., Newman, M.G., Shafir, T., and Tsachor, R. (2023). Unlocking the emotional world of visual media: An overview of the science, research, and impact of understanding emotion. Proc. IEEE *111*, 1–51. https://doi.org/10.1109/JPROC.2023.3273517.
4. Zhao, S., Jia, G., Yang, J., Ding, G., and Keutzer, K. (2021). Emotion recognition from multiple modalities: Fundamentals and methodologies. IEEE Signal Process. Mag. *38*, 59–73. https://doi.org/10.1109/MSP.2021.3106895.
5. Zhao, S., Yao, X., Yang, J., Jia, G., Ding, G., Chua, T.-S., Schuller, B.W., and Keutzer, K. (2022). Affective image content analysis: Two decades review and new perspectives. IEEE Trans. Pattern Anal. Mach. Intell. *44*, 6729–6751. https://doi.org/10.1109/TPAMI.2021.3094362.
6. Luo, Y., Ye, J., Adams, R.B., Li, J., Newman, M.G., and Wang, J.Z. (2020). ARBEE: Towards automated recognition of bodily expression of emotion in the wild. Int. J. Comput. Vis. *128*, 1–25. https://doi.org/10.1007/s11263-019-01215-y.
7. Bhattacharya, U., Mittal, T., Chandra, R., Randhavane, T., Bera, A., and Manocha, D. (2020). STEP: Spatial temporal graph convolutional networks for emotion perception from gaits. In Proc. AAAI Conf. Artif. Intell., *34* (AAAI Press), pp. 1342–1350. https://doi.org/10.1609/aaai.v34i02.5490.
8. Filntisis, P.P., Efthymiou, N., Potamianos, G., and Maragos, P. (2020). Emotion understanding in videos through body, context, and visual-semantic embedding loss. In Proc. European Conf. Computer Vision Workshops (Springer), pp. 747–755. https://doi.org/10.1007/978-3-030-66415-2_52.
9. Huang, Y., Wen, H., Qing, L., Jin, R., and Xiao, L. (2021). Emotion recognition based on body and context fusion in the wild. In Proc. IEEE/CVF Int. Conf. Computer Vision Workshops (IEEE), pp. 3609–3617. https://doi.org/10.1109/ICCVW54120.2021.00403.
10. Eleftheriadis, S., Rudovic, O., and Pantic, M. (2015). Multi-conditional latent variable model for joint facial action unit detection. In Proc. IEEE/CVF Int. Conf. Computer Vision (IEEE), pp. 3792–3800. https://doi.org/10.1109/ICCV.2015.432.
11. Fabian Benitez-Quiroz, C., Srinivasan, R., and Martinez, A.M. (2016). EmotioNet: An accurate, real-time algorithm for the automatic annotation of a million facial expressions in the wild. In Proc. IEEE/CVF Conf. Computer Vision and Pattern Recognition (IEEE), pp. 5562–5570. https://doi.org/10.1109/CVPR.2016.600.
12. Kollias, D., Cheng, S., Ververas, E., Kotsia, I., and Zafeiriou, S. (2020). Deep neural network augmentation: Generating faces for affect analysis. Int. J. Comput. Vis. *128*, 1455–1484. https://doi.org/10.1007/s11263-020-01304-3.
13. Chen, Y., and Joo, J. (2021). Understanding and mitigating annotation bias in facial expression recognition. In Proc. IEEE/CVF Int. Conf. Computer Vision (IEEE), pp. 14980–14991. https://doi.org/10.1109/ICCV48922.2021.01471.
14. Ruan, D., Mo, R., Yan, Y., Chen, S., Xue, J.-H., and Wang, H. (2022). Adaptive deep disturbance-disentangled learning for facial expression recognition. Int. J. Comput. Vis. *130*, 455–477. https://doi.org/10.1007/s11263-021-01556-7.
15. Aviezer, H., Trope, Y., and Todorov, A. (2012). Body cues, not facial expressions, discriminate between intense positive and negative emotions. Sciences *338*, 1225–1229. https://doi.org/10.1126/science.1224313.
16. Camurri, A., Lagerlöf, I., and Volpe, G. (2003). Recognizing emotion from dance movement: comparison of spectator recognition and automated techniques. Int. J. Hum. Comput. *59*, 213–225. https://doi.org/10.1016/S1071-5819(03)00050-8.
17. Dance Project Contributors (2020). European Union H2020 ICT Dance Project. http://dance.dibris.unige.it.
18. Niewiadomski, R., Mancini, M., Cera, A., Piana, S., Canepa, C., and Camurri, A. (2019). Does embodied training improve the recognition of mid-level expressive movement qualities sonification? J. Multimodal User Interfaces *13*, 191–203. https://doi.org/10.1007/s12193-018-0284-0.
19. Piana, S., Staglianò, A., Odone, F., and Camurri, A. (2016). Adaptive body gesture representation for automatic emotion recognition. ACM Trans. Interact. Intell. Syst. *6*, 1–31. https://doi.org/10.1145/2818740.
20. Beyan, C., Karumuri, S., Volpe, G., Camurri, A., and Niewiadomski, R. (2023). Modeling multiple temporal scales of full-body movements for emotion classification. IEEE Trans. Affect. Comput. *14*, 1070–1081. https://doi.org/10.1109/TAFFC.2021.3095425.
21. EnTimeMent Project Contributors (2020). The European FET PROACTIVE Project EnTimeMent. https://entimement.dibris.unige.it.
22. Olugbade, T., Bieńkiewicz, M., Barbareschi, G., D'amato, V., Oneto, L., Camurri, A., Holloway, C., Björkman, M., Keller, P., Clayton, M., et al. (2022). Human movement datasets: An interdisciplinary scoping review. ACM Comput. Surv. *55*, 1–29. https://doi.org/10.1145/3534970.
23. Gu, C., Sun, C., Ross, D.A., Vondrick, C., Pantofaru, C., Li, Y., Vijayanarasimhan, S., Toderici, G., Ricco, S., Sukthankar, R., et al. (2018). AVA: A video dataset of spatio-temporally localized atomic visual actions. In Proc. IEEE/CVF Conf. Computer Vision and Pattern Recognition (IEEE), pp. 6047–6056. https://doi.org/10.1109/CVPR.2018.00633.
24. Shafir, T., Tsachor, R.P., and Welch, K.B. (2015). Emotion regulation through movement: unique sets of movement characteristics are associated with and enhance basic emotions. Front. Psychol. *6*, 2030. https://doi.org/10.3389/fpsyg.2015.02030.
25. Melzer, A., Shafir, T., and Tsachor, R.P. (2019). How do we recognize emotion from movement? specific motor components contribute to the recognition of each emotion. Front. Psychol. *10*, 1389. https://doi.org/10.3389/fpsyg.2019.01389.
26. van Geest, J., Samaritter, R., and van Hooren, S. (2020). Move and be moved: The effect of moving specific movement elements on the experience of happiness. Front. Psychol. *11*, 579518. https://doi.org/10.3389/fpsyg.2020.579518.
27. Ajili, I., Mallem, M., and Didier, J.-Y. (2019). Human motions and emotions recognition inspired by lma qualities. Vis. Comput. *35*, 1411–1426. https://doi.org/10.1007/s00371-018-01619-w.
28. Liu, Z., Ning, J., Cao, Y., Wei, Y., Zhang, Z., Lin, S., and Hu, H. (2022). Video Swin Transformer. In Proc. IEEE/CVF Conf. Computer Vision and Pattern Recognition (IEEE), pp. 3202–3211. https://doi.org/10.1109/CVPR52688.2022.00320.
29. Wang, L., Xiong, Y., Wang, Z., Qiao, Y., Lin, D., Tang, X., and Van Gool, L. (2019). Temporal segment networks for action recognition in videos. IEEE Trans. Pattern Anal. Mach. Intell. *41*, 2740–2755. https://doi.org/10.1109/TPAMI.2018.2868668.

30. Feichtenhofer, C., Fan, H., Malik, J., and He, K. (2019). Slowfast networks for video recognition. In Proc. IEEE/CVF Int. Conf. Computer Vision (IEEE), pp. 6202–6211. https://doi.org/10.1109/ICCV.2019.00630.

31. Duan, H., Zhao, Y., Chen, K., Lin, D., and Dai, B. (2022). Revisiting skeleton-based action recognition. In Proc. IEEE/CVF Conf. Computer Vision and Pattern Recognition (IEEE), pp. 2969–2978. https://doi.org/10.1109/CVPR52688.2022.00298.

32. Cao, Z., Simon, T., Wei, S.-E., and Sheikh, Y. (2017). Realtime multi-person 2d pose estimation using part affinity fields. In Proc. IEEE/CVF Conf. Computer Vision and Pattern Recognition (IEEE), pp. 7291–7299. https://doi.org/10.1109/CVPR.2017.143.

33. He, K., Zhang, X., Ren, S., and Sun, J. (2016). Deep residual learning for image recognition. In Proc. IEEE/CVF Conf. Computer Vision and Pattern Recognition (IEEE), pp. 770–778. https://doi.org/10.1109/CVPR.2016.90.

34. Liu, Z., Lin, Y., Cao, Y., Hu, H., Wei, Y., Zhang, Z., Lin, S., and Guo, B. (2021). Swin transformer: Hierarchical vision transformer using shifted windows. In Proc. IEEE/CVF Int. Conf. Computer Vision (IEEE), pp. 10012–10022. https://doi.org/10.1109/ICCV48922.2021.00986.

35. MMAction2 Contributors (2020). OpenMMLab's Next Generation Video Understanding Toolbox and Benchmark. https://github.com/open-mmlab/mmaction2.

36. Deng, J., Dong, W., Socher, R., Li, L.-J., Li, K., and Fei-Fei, L. (2009). ImageNet: A large-scale hierarchical image database. In Proc. IEEE/CVF Conf. Computer Vision and Pattern Recognition (IEEE), pp. 248–255. https://doi.org/10.1109/CVPR.2009.5206848.

37. Kay, W., Carreira, J., Simonyan, K., Zhang, B., Hillier, C., Vijayanarasimhan, S., Viola, F., Green, T., Back, T., Natsev, P., et al. (2017). The kinetics human action video dataset. Preprint at arXiv. https://doi.org/10.48550/arXiv.1705.06950.

38. Pikoulis, I., Filntisis, P.P., and Maragos, P. (2021). Leveraging semantic scene characteristics and multi-stream convolutional architectures in a contextual approach for video-based visual emotion recognition in the wild. In Proc. IEEE Int. Conf. Automatic Face and Gesture Recognition (IEEE), pp. 01–08. https://doi.org/10.1109/FG52635.2021.9666957.

39. Carreira, J., and Zisserman, A. (2017). Quo vadis, action recognition? a new model and the kinetics dataset. In Proc. IEEE/CVF Conf. Computer Vision and Pattern Recognition (IEEE), pp. 6299–6308. https://doi.org/10.1109/CVPR.2017.502.

40. Yan, S., Xiong, Y., and Lin, D. (2018). Spatial temporal graph convolutional networks for skeleton-based action recognition. In Proc. AAAI Conference on Artificial Intelligence, *32* (AAAI Press), pp. 7444–7452. https://doi.org/10.1609/aaai.v32i1.12328.

41. AVA Dataset Contributors (2018). AVA Actions Dataset. https://github.com/cvdfoundation/ava-dataset.

42. Potapov, D., Douze, M., Harchaoui, Z., and Schmid, C. (2014). Category-specific video summarization. In Proc. European Conf. Computer Vision (Springer), pp. 540–555. https://doi.org/10.1007/978-3-319-10599-4_35.

43. Wu, C., Chen, Y., Luo, J., Su, C.-C., Dawane, A., Hanzra, B., Deng, Z., Liu, B., Wang, J.Z., and Kuo, C.-h. (2020). MEBOW: Monocular estimation of body orientation in the wild. In Proc. IEEE/CVF Conf. Computer Vision and Pattern Recognition (IEEE), pp. 3451–3461. https://doi.org/10.1109/CVPR42600.2020.00351.

Patterns, Volume *4*

Supplemental information

Bodily expressed emotion understanding through integrating Laban movement analysis

Chenyan Wu, Dolzodmaa Davaasuren, Tal Shafir, Rachelle Tsachor, and James Z. Wang

Table S1

AP values for all emotion categories on the BoLD validation set

To provide a comprehensive view of how LMA improves BEEU across all emotion categories, we present a category-specific performance analysis of MANet in this table. Consistent with Tables 8 and 9 in the main body of the text, "Baseline-1" represents the original V-Swin model without the dual-branch and fusion elements, essentially operating without the assistance of LMA labels. "Baseline-2" refers to the MANet model excluding the Bridge loss. Our final model is denoted as "Ours". For each emotion category, the most effective and second most effective approaches are highlighted with color indicators. The table illustrates that, out of 26 categories, our model outperforms in 16. Even in the remaining categories, our model's performance is not substantially inferior to either Baseline-1 or Baseline-2. Consequently, it can be inferred that integrating LMA labels from the BoME dataset and utilizing our proposed network model not only amplifies the accuracy of predicting happiness and sadness, as stated in the main text, but also significantly improves the prediction of various other emotions with negligible adverse effects on the remaining categories.

Emotion Category	Baseline-1	Baseline-2	Ours
Peace	27.59	29.73	30.49
Affection	30.69	30.11	33.16
Esteem	17.43	16.08	15.22
Anticipation	29.51	29.59	28.86
Engagement	44.67	46.57	44.35
Confidence	41.22	43.68	44.04
Happiness	44.97	44.41	45.76
Pleasure	28.12	31.56	30.11
Excitement	30.36	28.62	30.77
Surprise	11.53	13.24	14.89
Sympathy	11.49	13.36	16.42
Confusion	23.44	22.93	22.49
Disconnection	10.11	10.22	10.63
Fatigue	16.89	14.61	17.21
Embarrassment	3.22	3.55	3.80
Yearning	7.70	3.49	3.82
Disapproval	14.41	17.33	17.92
Aversion	10.00	10.73	9.54
Annoyance	18.81	20.48	19.44
Anger	14.90	15.15	14.00
Sensitivity	10.75	11.40	14.08
Sadness	17.91	17.60	26.66
Disquietment	21.94	22.53	21.06
Fear	17.09	17.46	18.49
Pain	4.44	4.61	5.97
Suffering	10.00	12.08	13.26
Mean	19.97	20.43	21.25
